# Evaluating the use of a CpG free promoter for long-term recombinant protein expression stability in Chinese hamster ovary cells

**DOI:** 10.1186/s12896-016-0300-y

**Published:** 2016-10-18

**Authors:** Steven C. L. Ho, Esther Y. C. Koh, Benjamin P. C. Soo, Mariati, Sheng-Hao Chao, Yuansheng Yang

**Affiliations:** 1 Bioprocessing Technology Institute, Agency for Science, Technology and Research (A*STAR), 20 Biopolis Way, #06-01 Centros, Singapore, 138668 Singapore; 2 Department of Microbiology, National University of Singapore, Block MD4, 5 Science Drive 2, Singapore, 117597 Singapore

**Keywords:** Recombinant protein expression, CHO cells, Gene silencing, DNA methylation, Histone modifications

## Abstract

**Background:**

Methylated CpG dinucleotides in promoters are associated with the loss of gene expression in recombinant Chinese hamster ovary (CHO) cells during large-scale commercial manufacturing. We evaluated a promoter devoid of CpG dinucleotides, CpGfree, in parallel with a similar CpG containing promoter, CpGrich, for their ability to maintain the expression of recombinant enhanced green fluorescent protein (EGFP) after 8 weeks of culturing.

**Results:**

While the promoters gave similar transient expression levels, CpGfree clones had significantly higher average stable expression possibly due to increased resistance to early silencing during integration into the chromosome. A greater proportion of cells in clones generated using the CpGfree promoter were still expressing detectable levels of EGFP after 8 weeks but the relative expression levels measured at week 8 to those measured at week 0 did not improve compared to clones generated using the CpGrich promoter. Chromatin immunoprecipitation assays indicated that the repression of the CpGfree promoter was likely linked to histone deacetylation and methylation. Use of histone deacetylase inhibitors also managed to recover some of the lost expression.

**Conclusion:**

Using a promoter without CpG dinucleotides could mitigate the early gene silencing but did not improve longer-term expression stability as silencing due to histone modifications could still take place. The results presented here would aid in promoter selection and design for improved protein production in CHO and other mammalian cells.

## Background

Recombinant therapeutic proteins such as monoclonal antibodies are currently used to treat various cancers and autoimmune diseases. Chinese hamster ovary (CHO) cells transfected with plasmid vectors carrying the required gene are used to produce some of these recombinant products [1, 2]. Loss of recombinant gene expression in transfected CHO cells during long-term culture is commonly reported and is a major concern during production [3–6]. Any significant loss of productivity during the production process can affect both product yield and quality [7]. It is also preferred that cell lines are able to maintain recombinant protein expression without the need to supplement any selection reagent as these reagents are toxic and costly. Expression levels of the protein are expected to remain comparable to the start of culturing after the entire scale up and production process, retaining at least 70 % of initial levels for the clone to be considered stable [8].

One reason for the drop in expression is the gradual loss of gene copies during long-term culture resulting in decreased transcripts and thus the recombinant protein level [9–11]. This loss of gene copies had been linked to the inherent genetic instability of the recombinant CHO cell lines [6]. There are also reports of recombinant CHO cell lines losing protein expression levels without losing gene copies when the transcripts decrease due to transcriptional silencing [3]. The high number of gene copies integrated into the chromosome of high producing cell lines can result in repeat-induced gene silencing [12]. Transcriptional silencing is also linked to methylated cytosine on the CpG dinucleotides of promoters in recombinant protein producing CHO cells [4, 13–15]. CpGs are interesting, small DNA moieties which can be easily interspersed within DNA sequences to exert significant regulatory effect on gene expression [16]. CpG is methylated by DNA methyltransferases (DNMT) and the process silences genes by directly inhibiting transcription activation through disrupting the binding of transcription factors [17–19]. Methylated CpGs can also interact and recruit proteins that repress gene expression. Proteins with methyl-CpG binding domains (MBD) like MeCP2 can recruit either co-repressors or chromatin modifying enzymes like histone deacetylases (HDAC) [15, 20]. As maintaining transgene expression level is important to many applications, several solutions to reduce the effects of gene silencing due to CpG methylation and improve expression stability have been proposed.

A possible solution is to include epigenetic regulatory DNA elements which are able to modify the chromatin structure and aid in maintaining an open chromatin structure for gene expression [21]. Use of DNA regulatory elements like the locus control regions (LCR), matrix attachment regions (MAR) [22–24], insulators [25], CpG island elements (IE) [26] and ubiquitous chromatin opening elements (UCOE) [27, 28] have been discussed in reviews [21, 29]. Another possible solution is to supplement the culture media with DNMT inhibitors to delay or reverse DNA methylation to maintain expression [13, 30]. This can be hard to implement as the chemicals could be toxic and the transient effects are reversed once the chemical is removed. We could also maintain expression by keeping the selection pressure used to identify positive transfectants. Gene expression can also be maintained by supplementing the selection drug throughout culture period. Studies have shown that under selective conditions, integrated CpG rich reporters were not susceptible to methylation [31]. Care has to be taken when using media additives as some additives could complicate downstream processes and add costs to production [32]. Another solution could be to prevent CpG methylation from taking place by using promoters free of CpG. CpG free promoters and plasmids have been commonly adopted in gene therapy to reduce inflammation and methylation related loss of expression [33]. We had also previously observed that promoters with lower CpG counts exhibited greater expression stability [23]. It is still unclear how CpG free promoters would perform at improving long-term recombinant gene expression stability in transfected CHO cells.

In this study, we compared two promoters which comprised of the same enhancers and intron regions, and core promoters from a similar source with the main difference being that one promoter is free of CpG dinucleotides. The promoters were used to drive the expression of a recombinant enhanced green fluorescent protein gene (EGFP) in CHO cells. The promoters had similar transient expression levels but the CpG free promoter had significantly higher stable expression levels. Stability of the EGFP expression was evaluated after 8 weeks of passaging without any selection pressure. Interestingly, CpGfree promoter increased the proportion of cells expressing detectable levels of EGFP in clones after 8 weeks but not the relative expression levels compared to the CpGrich promoter. Subsequent chromatin immunoprecipitation (ChIP) analysis revealed similar gene repressing histone modifications in unstable clones from both the CpGfree and CpGrich promoters.

## Methods

### Vector construction

**Fig. 1:**
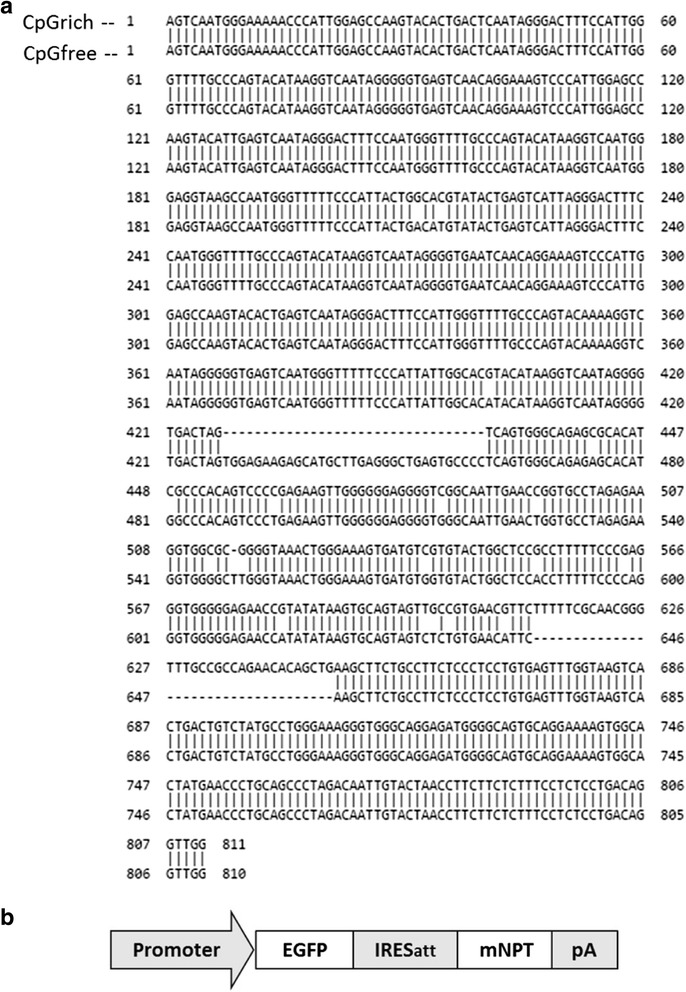
Promoter sequences and schematic representation of the vector used in the study. **a** Sequence of the CpGrich (top strand) and CpGfree (bottom strand) promoters used in the study. Both promoters were cloned from vectors from InvivoGen. The promoters have 90 % similarity and alignment was done online using nucleotide BLAST at http://blast.ncbi.nlm.nih.gov/Blast.cgi. **b** Structure of the expression cassette used to express an enhanced green fluorescence protein (EGFP) and a mutant neomycin phosphotransferase selection marker with amino acid substitution D261G (mNPT)

The promoters used in this study combined the mouse cytomegalovirus (CMV) enhancer, the human elongation factor 1 alpha core promoter and a synthetic intron at the 5′ untranslated region (UTR). CpG carrying versions of the promoter was cloned from the pCpGrich-mcs vector (InvivoGen, San Diego, CA) and the CpG free version was cloned from pCpGfree-mcs (InvivoGen). The promoters were labelled as CpGrich and CpGfree respectively and the sequences are listed in Fig. 1a. The cloned promoters were used to replace the human CMV promoter of a bicistronic vector for expressing EGFP used in a previous study to generate the vectors CpGrich and CpGfree [23] (Fig. 1b). An attenuated internal ribosome entry site (IRESatt) and mutant neomycin phosphotransferase (mNPT) was used to increase the stringency of selection for high producing cell lines [34–36].

### Cell culture and transfection

CHO K1 cells (American Type Culture Collection, Manassas, VA) were cultured in tissue culture plates using Dulbecco’s modified Eagle’s medium (DMEM) + GlutaMax^TM^ (Life Technologies) supplemented with 10 % fetal bovine serum (FBS) (Sigma-Aldrich, St. Louis, MO). Cells were passaged every 3 to 4 days by diluting cells to 2 × 10^5^ cells/mL. Cell density and viability were measured using the trypan blue exclusion method on a Vi-cell XR cell viability analyzer (Beckman Coulter, CA).

Three separate transfections were performed using each promoter using the Nucleofector I system from Lonza (Cologne, Germany). 5 × 10^6^ cells were transfected with 5 μg of linearized plasmids in each transfection. The transfected cells were transferred to 6-well tissue culture plates containing DMEM supplemented with 800 μg/mL of G418 (Sigma-Aldrich) for selection 24 h after transfection. Fluorescence-activated cell sorting (FACS) analysis was also performed using FACS Calibur (Becton Dickinson, Franklin Lakes, NJ) to determine the transient expression obtained using each promoter. Upon recovery of the stably transfected pools, at least nine clones were randomly selected from each stable pool by limiting dilution for a total of 30 clones to be carried forward for stability tracking.

### Stability tracking for stably transfected clones

Clones were subsequently cultured in 6-well tissue culture plates in the absence of G418 for 8 weeks. Mean fluorescent intensity (MFI) for each clone before (week 0) and after (week 8) stability testing were measured with a FACS Calibur (Becton Dickinson). Banked cells of week 0 clones were thawed and EGFP expression quantified in parallel to ensure differences were not due to drifts in the system’s sensors. EGFP expression retained for each clone was calculated as the ratio of MFI of the clone measured at the end of stability testing to the intensity at the start of stability testing. Detailed protocols for stability testing performed had been previously reported [26]. Cells pellets were also collected at the start and end of stability testing after centrifuging at 100 × g for subsequent molecular analysis.

### Determining gene copy and mRNA levels

Genomic DNA and total RNA were isolated from 5 × 10^6^ cells using the Gentra Puregene Cell Kit (Qiagen, Hilden, Germany) and the RNAqueous-4PCR kit (Ambion, Austin, TX), respectively. The extracted RNA was used to generate first-strand cDNA using ImProm-II Reverse Transcription System (Promega, Madison, WI). Relative EGFP gene copy numbers and mRNA levels were determined by using real-time quantitative PCR as described previously [3]. β-actin and eukaryotic translation elongation factor-1 alpha-1 (EF1α1) served as the internal controls to normalize the variation in input amount and quality of DNA and mRNA respectively. Primer pairs used are as follows: EGFP-forward (5′-CAAGCAGAAGAACGGCATCAA-3′) and EGFP-reverse (5′- GGACTGGGTGCTCAGGTAGTG-3′), β-actin-forward (5′ AGCTGAGAGGGAAATTGTGCG-3′) and β-actin-reverse (5′- GCAACGGAACCGCTCATT-3′), EF1α1-forward (5′- TGGAAGATGGCCCTAAATTC-3′) and EF1α1-reverse (5′- AACGACCCAGTGGAGGATAG-3′).

### ChIP for methylated DNA and chromatin modifications

ChIP assays were carried out using EZ ChIP kit (Millipore) according to a modified process based on the manufacturer’s protocol. DNA fragments sized at between 200 and 1000 bp were obtained by sonication using a Microson Ultrasonic Cell Disruptor (Misonix, Farmingdale, NY). The antibodies used for IP were anti-5-methylcytosine (anti-5-mC), anti-histone H3 acetyl-lysine 9 (anti-H3K9Ac), anti-histone H3 trimethyl-lysine 9 (anti-H3K9Me3) and anti-heterochromatin protein 1 (anti-HP1) (all from Abcam, Cambridge, MA). Antibodies were added to the sheared chromatin individually and incubated at 4 °C overnight. The DNA/protein/antibody complex was then pulled down by protein G agarose and the DNA in the complex was purified using QIAquick PCR purification kit (Qiagen). Real-time quantitative PCR was performed to determine the relative amount of DNA that was immunoprecipitated by each antibody in week 0 and week 8 samples. DNA enriched using anti-5-mC and anti-HP1 antibodies were quantified using primer pairs specific to the promoters and DNA enriched using anti-H3K9Me3 and anti-H3K9Ac used primers specific to the EGFP gene. The primer pairs used to amplify the CpGrich promoter, CpGfree wild type promoter and EGFP genes include: CpGrich promoter (5′-GACTAGTCAGTGGGCAGAGC-3′ and 5′-ACCCGTTGCGAAAAAGAACG-3′), CpGfree promoter (5′-GAAGAGCATGCTTGAGGGCT-3′ and 5′-TTTACCCAAGCCCCACCTTC-3′) and EGFP (5′- TACCAGCAGAACACCCCCAT -3′ and 5′- ACCATGTGATCGCGCTTCTC -3′).

### DNA methyltransferase and histone deacetylase inhibitor treatment

Selected stable and unstable clones generated using both promoters were seeded into 24-well plates at cell density of 3 × 10^5^ cells/mL and cultured for 24 h in selection free DMEM. Media was changed to fresh DMEM containing either 4 μM 5-Aza-2′-deoxycitidine (5-Aza-dC, Sigma-Aldrich), a DNMT inhibitor, or 2 mM sodium butyrate (NaBu, Sigma-Aldrich), a HDAC inhibitor, or without inhibitors as a control. Cells were then further cultured in the DMEM supplemented with inhibitors for 24 h before being harvested for FACS analysis to obtain the MFI of the clones after treatment.

## Results

### Generating clones and testing EGFP expression level and stability

**Fig. 2:**
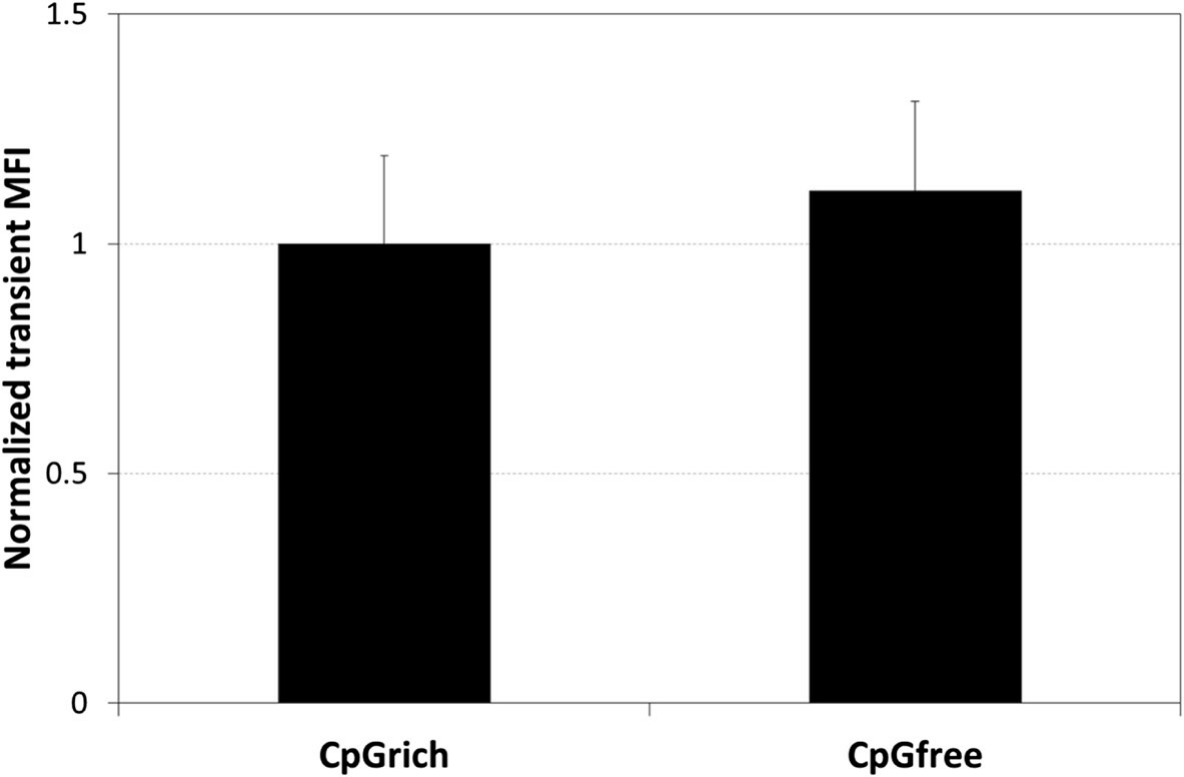
Comparing transient EGFP expression level obtained using the CpGrich and CpGfree promoters. Cells were transfected and EGFP expression levels measured using a flow cytometer after 24 h. Each bar and standard deviation represents the results obtained from duplicate experiments of three separately transfected pools. Results were normalized for transfection efficiency for each sample before normalizing to CpGrich expression level

**Fig. 3:**
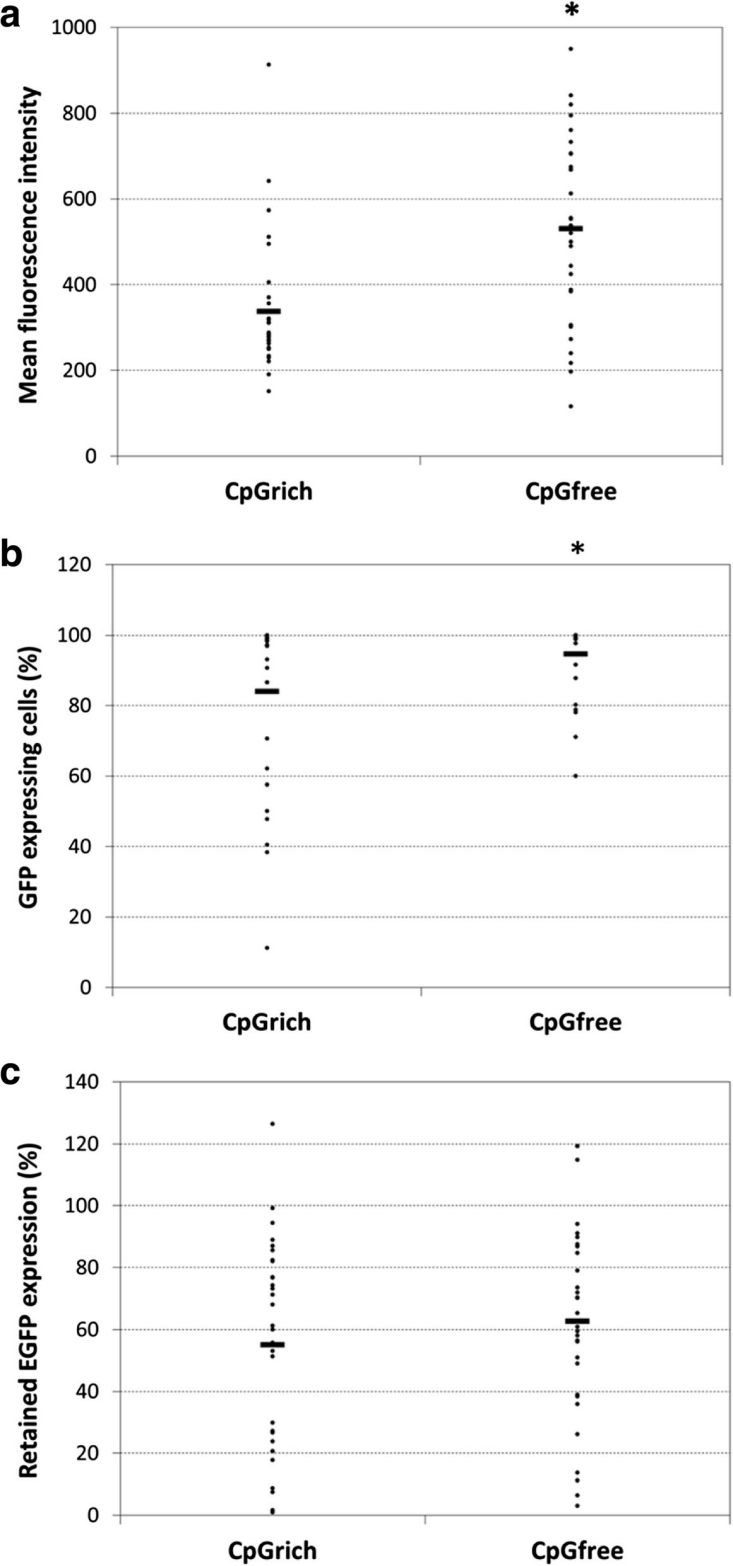
Comparing EGFP expressing level and stability of clones using the CpGrich and CpGfree promoters. **a** Mean fluorescence intensity (MFI) of 30 stably transfected clones for each promoter at week 0 before selection pressure was removed at the start of stability testing. Stability testing was subsequently performed by culturing the cells in selection free media for 8 weeks. The clones were randomly isolated from three separately generated pools. **b** Percentage of EGFP expressing cells after 8 weeks stability testing. **c** Retained EGFP expression after 8 weeks stability testing. Each point represents the MFI of an individual clone and the horizontal bar represents the average MFI of the 30 clones. Values of CpGfree clones which are statistically different from CpGrich are indicated by an *(*p* < 0.05)

Transient expression using the two promoters, CpGrich and CpGfree, were first compared. EGFP expression using CpGfree was around 10 % higher than CpGrich but the difference was not significant after normalizing for transfection efficiency (Fig. 2). After confirming similarity in the promoter strengths, we generated three stably transfected pools separately for each promoter. The CpGfree pools recovered (reached 80 % confluence) one passage faster than the CpGrich pools. Heterogeneous pools were not suitable for stability testing in our study as any loss of recombinant protein expression in pools could be due to clones with low expression level outgrowing the rest of the population instead of molecular reasons like DNA methylation. Upon recovery, we randomly selected at least nine clones from each pool to obtain a total of 30 stably transfected clonal cell lines for each promoter. We checked that the entire population of each clone was expressing EGFP at the start of stability testing, week 0. EGFP expression was higher for the CpGfree clones with an average MFI of 530 compared to 336 for the CpGrich clones (Fig. 3a). CpGfree stable pools also had higher expression levels than CpGrich (data not shown). The highest and lowest EGFP expressing clones were similar for both promoters at MFI of around 900 and 150. These MFI readings were recorded as the EGFP expression at week 0 for the clones. These two sets of clones were subsequently passaged for 8 weeks without any selection reagent before EGFP expression was measured again.

Expression stability was measured using two values, the percentage of cells within the clonal population still expressing EGFP levels above that of non-transfected CHO K1 cells and the retention of EGFP expression levels compared to week 0 after 8 weeks of culture. The CpGrich clones had 83.9 % of the population retaining EGFP population on average (Fig. 3b). 21 out of the 30 clones still had more than 90 % of the population expressing detectable levels of EGFP and the worst performing clone had only 11.1 % of the population with EGFP expression. CpGfree clones performed better, averaging 94.6 % of the cells staying positive for EGFP expression after 8 weeks. The worst performing clone still had 60 % of the population expressing the recombinant protein.

The EGFP expression retained was determined as a ratio of the expression level at week 8 to the level at week 0. There was no significant difference between the two promoters for their ability to maintain gene expression over the 8 weeks. The CpGrich clones retained an average of 55 % of EGFP expression while the CpGfree clones performed slightly better at 62 % (Fig. 3c). 13 of the 30 CpGrich clones still had expression above 70 % of their initial expression and 14 of the 30 clones using CpGfree achieving the same result. Our results indicated that using CpGfree might be beneficial during the drug selection process but does not provide any significant improvements to long-term expression stability. Due to a higher initial expression level, the final recombinant protein levels were still higher despite no improvements to stability. Quantitative PCR and ChiP assays were subsequently performed to understand if the loss of expression was due to changes in gene copies or other gene silencing related molecular mechanisms.

### Gene copy and mRNA levels of the clones before and after stability testing

To understand why the CpGfree promoter did not have a greater effect on improving stability, nine clones exhibiting a range of retained expression were chosen for both CpGrich and CpGfree promoters for gene copy and mRNA level analysis. The clones ranged from retaining only 1 % EGFP expression to those which maintained the same expression level after the eight weeks.

**Fig. 4:**
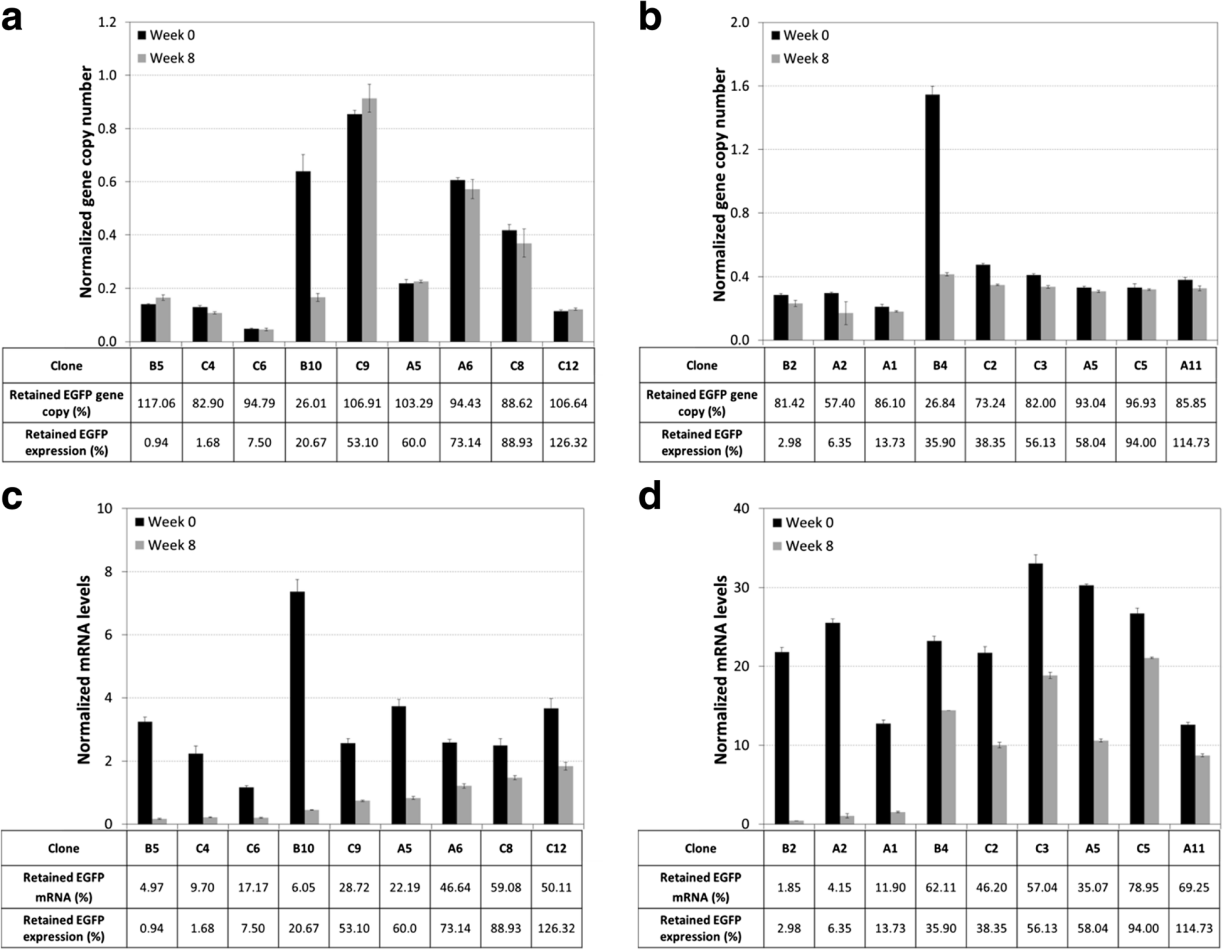
Comparing relative EGFP gene copies and mRNA levels. EGFP Gene copies were determined for (**a**) CpGrich and (**b**) CpGfree (**b**) clones and relative EGFP mRNA levels for (**c**) CpGrich and (**d**) CpGfree clones at week 0 and week 8. β-actin and eukaryotic translation elongation factor-1 alpha-1 (EF-1α) served as the internal controls to normalize the variation in input amount and quality of DNA and mRNA respectively. Retained EGFP expression level, gene copy and mRNA values in the tables were calculated as the ratios of their values at the end of stability testing at week 8 to their starting levels at week 0. Black bar represents values for week 0 and the grey bar represents week 8. Each point and standard deviation represents the average derived from replicated measurements of two separately prepared samples

Gene copy number remained similar for most CpGrich clones and stayed above 80 % with only CpGrich-B10 exhibiting a drop to 26 % after the eight weeks (Fig. 4a). Most of the CpGfree clones also maintained their gene copy numbers and only one clone, CpGfree-B4, dropped to only 27 % of initial gene copies (Fig. 4b). There was little correlation between the change in gene copies and the retained EGFP expression levels for both promoters (*R* ^*2*^ = 0.355 for CpGrich and *R* ^*2*^ = 0.152 for CpGfree). We next looked at the mRNA levels before and after stability testing for the same set of nine clones from each promoter. mRNA levels generally correlated better to the changes in EGFP expression levels. mRNA levels decreased to between 5 and 59 % of the starting levels at week 0 after stability testing for the CpGrich clones (Fig. 4c). CpGrich-B5 which retained the least expression also retained the lowest mRNA level. mRNA levels for some of the clones which maintained EGFP expression, like clone CpGrich-C12, still decreased to 50 %. The change in mRNA levels correlated better with the change in EGFP expression levels as compared to the gene copy data (*R* ^*2*^ = 0.836). The only exception was CpGrich-B10 which had a larger than expected drop in mRNA levels to only 6 % while maintaining 20 % expression, possibly due to the large drop in the gene copy number described earlier. Changes to the mRNA expression level for the CpGfree clones also correlated better to the retained EGFP expression (*R* ^*2*^ = 0.737) (Fig. 4d). Clone CpGfree-B2 which retained 2.98 % EGFP expression also retained the lowest mRNA level at 1.85 %. Both of the stable clones CpGfree-C5 and -A11 which retained 94 % and 114 % EGFP expression also retained the most mRNA levels of 78.9 % and 69.3 % (Fig. 4d).

Our attempts to improve expression stability for a recombinant protein using a CpGfree promoter did not yield significant improvement. It was interesting that the CpGfree promoter which had no CpG sites available for DNA methylation did not perform much better than the CpGrich promoter. We selected two unstable clones which maintained gene copies (>80 %) but exhibited both decreased mRNA levels and expression levels, and one stable clone which maintained EGFP expression level (>90 %) for further ChIP studies.

### ChIP analysis of the clones before and after stability testing

**Fig. 5:**
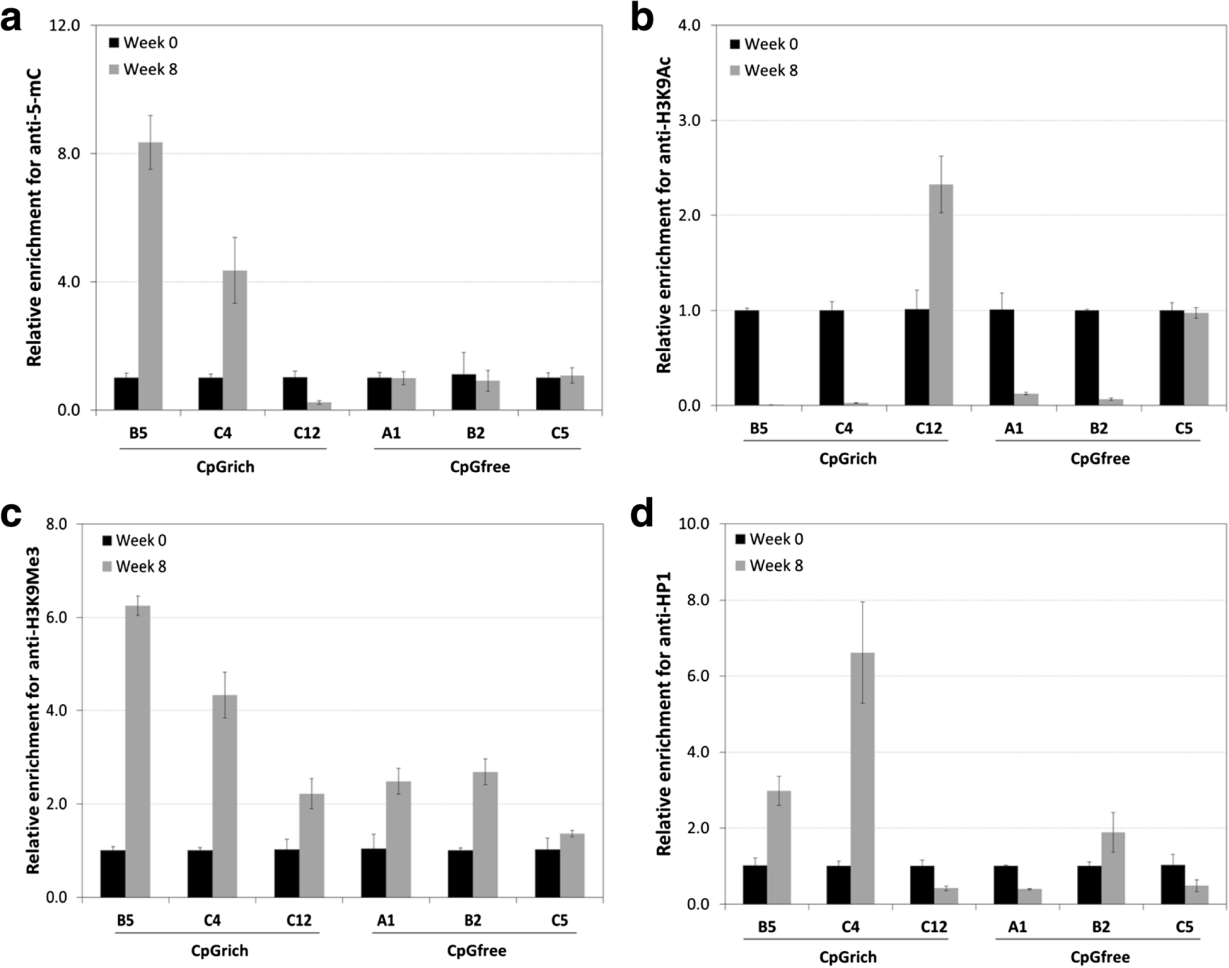
Comparing DNA methylation and histone modifications for unstable and stable clones for CpGfree and CpGrich promoters by ChIP assays. DNA was extracted from samples of two unstable clones and one stable clone for each promoter and immunoprecipitation performed using four different antibodies: (**a**) anti-5-mC, (**b**) anti-H3K9Ac, (**c**) anti-H3K9Me3 and (**d**) anti-HP1. Enrichment was measured by real-time quantitative PCR. Black and grey bars represent data for samples from week 0 and from week 8 respectively. Results were normalized to their week 0 results and each bar represents triplicate measurements of two separate sets of experiments

Clones B5, C4 for CpGrich and clones A1, B2 for the CpGfree were selected as the unstable clones. The stable clones selected for analysis were C12 and C5 for the CpGrich and CpGfree promoters respectively. All of the clones maintained their gene copies during stability testing. ChIP was performed separately using four antibodies anti-5-mC, anti-H3K9Ac, anti-H3K9Me3, and anti-HP1 on both the week 0 and week 8 samples for the selected clones. Methylated cytosines on CpG dinucleotides which are common markers for unstable CHO cell lines were first targeted using the anti-5-mC antibodies for precipitation. CpGrich clones B5 and C4 with EGFP retention of only around 1 % was enriched by 8-fold and 4-fold respectively when comparing their week 8 with week 0 samples (Fig. 5a). The stable CpGrich-C12 which maintained expression above 100 % was instead depleted at week 8. No enrichment was observed for all the three CpGfree clones analyzed A1, B2 and C5, which was consistent with the expectation that the CpGfree promoter has no CpG sites for methylation.

Analysis was next performed for the major histone modification markers of histone H3 lysine9 (H3K9) acetylation and trimethylation using the respective antibodies for enrichment. Acetylated histones favor open chromatin structures and correlate strongly with active gene transcription [37]. H3K9 acetylation decreased to below 0.1 for unstable clones of both the CpGrich and CpGfree clones (Fig. 5b). There was an enrichment of more than 2-fold for the stable CpGrich-C12 and no significant changes to the stable CpGfree-C5 clone. H3K9 trimethylation is commonly associated with loss of recombinant gene expression [38] and this was reflected in the unstable clones which had 2.5-fold to 6.2-fold enrichment for week 8 samples as compared to samples collected at the start of stability testing when using anti-H3K9Me3 antibodies (Fig. 5c). The stable clone was also enriched but at a lower level. Among the stable clones, CpGrich-C12 had 2.2-fold enrichment and CpGfree-C5 had 1.4-fold enrichment. Heterochromatin protein 1 (HP1) is a nucleosomal protein associated with gene repression associated with position effect variegation [39, 40]. When anti-HP1 antibody was used for enrichment, samples of CpGrich-B5 and -C4 at week 8 exhibited enrichment of 3-fold and 6.6-fold higher than samples from week 0 (Fig. 5d). Stable CpGrich-C12 was lower at week 8 at 0.4-fold of week 0 levels. While the stable CpGfree clone, C5, was also depleted at week 8 at about 0.5-fold, the unstable CpGfree clones gave contrasting results. CpGfee-A1 enriched using anti-HP1 was 0.4-fold of the starting levels while CpGrich-B2 was 1.9-fold higher than week 0.

### Effect of chemical treatment on restoring EGFP expression

**Fig. 6:**
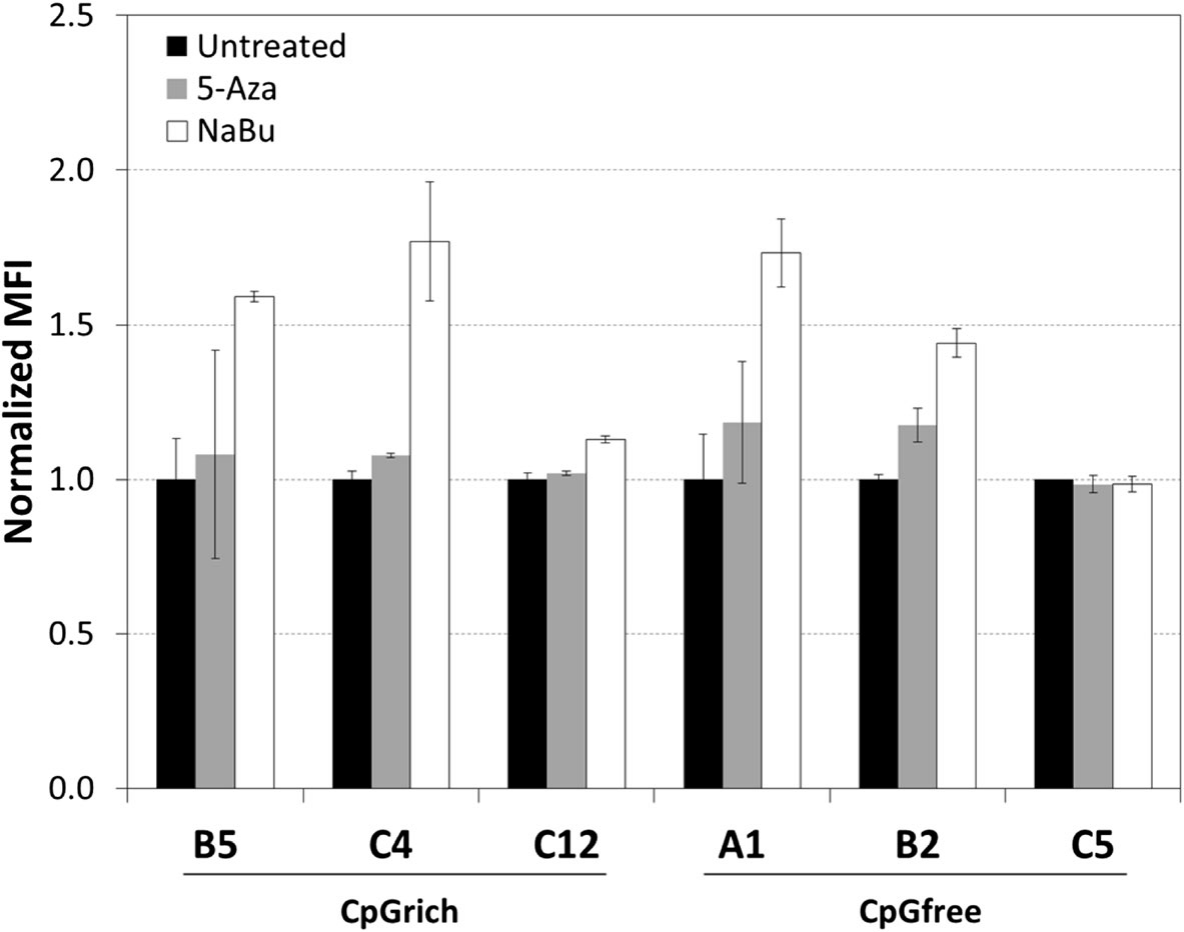
Restoring EGFP expression using DNA methyltransferase (DNMT) inhibitor, 5-Aza-2′-deoxycitidine (5-Aza-dC), and histone deacetylase (HDAC) inhibitor, sodium butyrate (NaBu). Clones generated using CpGrich (B5, C4 unstable clones and C12 stable clone) and CpGfree (A1,B2 unstable clones and C5 stable clone) at week 8, the end of stability testing, were treated with DNMT or HDAC inhibitors for 24 h before MFI was measured again. Black bars represent samples without treatment, grey bars for samples treated with 5-Aza-dC and white bars represent samples treated with NaBu. Each bar represents duplicate measurements of two independent experiments for each clone. MFI values were normalized to the untreated control

Week 8 samples of the clones used for ChIP analysis were treated with 5-Aza-dC, a DNMT inhibitor, and NaBu, a HDAC inhibitor. The cells were treated for 24 h before mean fluorescent intensity level of EGFP were analyzed by FACS. Treating the unstable cells using 5-Aza-dC did not result in any significant improvements in EGFP expression (Fig. 6). Using NaBu had greater effect and the EGFP levels for all the unstable clones improved around 1.5-fold compared to the untreated samples. Both the stable clones tested, CpGrich-C12 and CpGfree-C5, did not exhibit any significant changes. These results indicated that histone modifications play a more important role at silencing the gene expression during long term culture compared to DNA methylation.

## Discussion

Methylated CpG dinucleotides are commonly associated with loss of recombinant gene expression in CHO cell lines [4, 6, 13]. In this study, we evaluated the use of the promoter, CpGfree, which was devoid of CpG for recombinant protein production in CHO cells and compared it with CpGrich, a promoter with similar structure but contains CpG dinucleotides. Transient EGFP expression levels were similar but CpGrich had significantly lower expression in stably transfected cell lines. After 8 weeks of culturing in the absence of selection pressure for stability testing, CpGfree clones maintained a larger proportion of cells still expressing the recombinant protein. This greater proportion of protein expressing population did not translate to an actual higher protein expression level and we observed no significant difference in the retained EGFP levels when comparing the week 8 levels to the starting levels. The drop in gene expression was not a result of losing gene copies for most clones but instead decreased transcription leading to lower mRNA levels for both promoters. ChIP analysis revealed that the vector integrated on the chromosome for unstable CpGrich clones were enriched for 5-methylcytosine and H3K9 trimethylation, and depleted for H3K9 acetylation. The CpGfree clones which lost the majority of their expression also exhibited similar chromatin histone associations even without any methylated sites on the promoter. Gene silencing due to histone modifications appeared to reduce the benefits of using CpGfree to improve long-term recombinant protein expression stability in CHO cells. Histone modifications were also identified as better indicators of expression stability than CpG methylation and histone binding proteins like HP1. Treatment of the unstable clones using a HDAC inhibitor also yielded a more significant improvement of recombinant EGFP expression compared to a DNMT inhibitor. The DNMT inhibitor used, 5-Aza-dC, had been previously reported to have limited effect at restoring gene expression in CHO clones [13].

Transgenes have the potential to be silenced within days of integrating into the chromosome in applications involving CHO cells, stem cells and gene therapy in lung cells [12, 33, 41]. The transfected genes can also be targeted for repeat-induced gene silencing when a high number of gene copies are integrated for recombinant mammalian cell lines [12]. We had observed that the transient EGFP expression levels of the two promoters were similar but expression of stably transfected pools and clones after drug selection for CpGfree was significantly higher. The overall higher EGFP expression would also mean higher expression of the selection marker as the genes were linked by IRES and this was a possible reason why CpGfree pools recovered faster during selection. We had also previously observed that including expression augmenting elements which reduce the effects of gene silencing increased the number of colonies surviving drug selection without increasing recombinant gene expression [23]. The benefits of using CpGfree to possibly reduce early silencing upon vector integration include obtaining stable pools faster and being able to use higher selection drug concentrations for a more stringent selection process. While not determined in our study, other reports had indicated that integrated transgenes remain unmethylated during early stages of transfected CHO cell lines [13, 42, 43]. It is also possible that the higher early expression when using the CpGfree promoter could have arose due to differences in the factors recruited or chromatin modifications compared to the CpGrich promoter and future follow-up analysis could provide some interesting results.

While CpG methylation is linked to gene silencing, studies have indicated that methylation does not always initiate silencing but is possibly a downstream event after the gene gets silenced [20, 43]. We had expected that the CpGfree promoter to confer some benefits for sustained recombinant protein expression even if methylation was only a partial reason for loss of gene expression. Our studies yielded no significant benefits to long-term stable expression when using the CpGfree promoter as the EGFP expression retained was similar to the CpGrich promoter. Majority of the clones analyzed for both promoters did not lose gene copies but exhibited a drop in mRNA level, a phenomenon which had been reported by other studies as well [3, 44]. Any loss of gene copies could have arose during homologous recombination to repair DNA strand breaks [45]. Some clones maintained EGFP levels despite a drop in mRNA levels were possibly limited by translation rate or saturation of the intracellular protein levels. The CpGfree clones appeared to be more resistant to total silencing of the gene as seen by the larger proportion of the population still expressed detectable levels of EGFP. High density DNA methylation is required for stable epigenetic imprint for transfer of transcriptional repression [46]. Using CpGfree could have impeded the complete and stable silencing of transgenes, allowing cells to express EGFP for longer periods.

Both ChIP data and chemical inhibitor treatment of selected unstable clones linked the decreasing gene expression of the CpGfree promoter to chromatin modifications of increased H3K9 trimethylation and deacetylation. It had been shown that histone H3 hypoacetylation is linked to loss of recombinant gene expression using simian virus 40 (SV40) promoter driven vectors in CHO cells [47]. Another study performed using a CMV promoter to drive gene expression in CHO cells also observed that histone modifications were playing a role in regulating transgene expression during clone generation [42]. Similar loss of gene expression had been observed with a range of promoters in CHO cells, indicating that promoter choice which is commonly associated with expression stability might not be as important as we expected. Another interesting observation made was the depleted HP1 for the unstable CpGrich clone A1. There are reports of competitive binding by other proteins like DNMT3a displacing HP1 and could be the reason for lower HP1 in some samples [48]. The interactions and processes related to histone modifications still require more work to obtain a better understanding [38] and until then, proteins interacting with histone modifications might not be good indicators for expression stability. Screening of the promoters using TRANSFAC® software identified binding sites for factors like YY1 which can down-regulate gene expression and could be another possible reason for the failure of CpGfree at improving long-term stability [49, 50].

## Conclusion and future work

Long-term expression stability is arguably of greater importance to large scale production of protein therapeutics than many other applications of recombinant protein expression in mammalian cells due to the health and commercial implications. While the CpGfree promoter devoid of DNA methylation sites did not improve the percentage of expression retained, it could possibly still be useful during the early stages of generating recombinant CHO cell lines with its potential resistance to early gene silencing. The faster recovery during selection would help shorten the timeline required to generate stable pools. The higher initial titer from these stable pools would allow fast generation of more material using stably transfected pools for early assay development and laboratory testing. Our observation that a significantly larger proportion of cells were still producing the recombinant protein after prolonged passaging with the CpGfree promoter is not as that critical to therapeutics production where the main concern is the collective productivity level. It could still be important to cell engineering studies where high expression level is not as critical.

We observed that preventing CpG methylation alone does not prevent gradual gene silencing as histone modifications which repress recombinant gene expression still take place. Further analysis of histone modifications to identify other possible correlations could also aid in providing a clearer picture of the interactions which affect long-term gene expression in CHO cells. There had been attempts to identify recombinant CHO cell lines with long-term stable protein production using markers like DNA methylation hotspots on the promoter [4]. Based on our observations, histone modification markers might be better indicators of expression stability and also targets for engineering to improve long-term expression stability.

Our results could be promoter specific and the stability of other promoters could still benefit from removal of CpG dinucleotides. Our experience with removing CpGs from commonly used promoters with random mutations mostly yielded promoters with lower strengths. A systematic method to remove CpG dinucleotides without impairing the strength of the promoter would be required for more promoters and their CpG free versions need to be compared. Another area which could provide some improvements to expression stability is codon optimization. Current codon optimization for recombinant protein expression in CHO cells are focused more on expression level and not for sustained gene expression [51, 52]. Analysis of the protein sequences which are expressed at constant levels throughout long-term passaging of CHO cultures could provide some information on designing silencing-proof promoters and gene sequences. Follow up studies using site-specific integration and lentiviral transfection could provide added insights to the effects of integration site and copy number on using CpGfree promoters in CHO cells.

## Abbreviations

5-Aza-dC: 5-Aza-2′-deoxycitidine
anti-5-mC: Anti-5-methylcytosine
anti-H3K9Ac: Anti-histone H3 acetyl-lysine 9
anti-H3K9Me3: Anti-histone H3 trimethyl-lysine 9
anti-HP1: Anti-heterochromatin protein 1
ChIP: Chromatin immunoprecipitation
CHO: Chinese hamster ovary
CMV: Cytomegalovirus
DMEM: Dulbecco’s modified Eagle’s medium
DNMT: DNA methyltransferases
EF1α1: Eukaryotic translation elongation factor-1 alpha-1
EGFP: Enhanced green fluorescent protein
FACS: Fluorescence-activated cell sorting
FBS: Fetal bovine serum
H3K9: Histone H3 lysine9
HDAC: Histone deacetylases
HP1: Heterochromatin protein 1
IE: CpG island elements
IRESatt: Attenuated internal ribosome entry site
LCR: Locus control regions
MAR: Matrix attachment regions
MBD: Methyl-CpG binding domains
MFI: Mean fluorescent intensity
mNPT: Mutant neomycin phosphotransferase
NaBu: Sodium butyrate
SV40: Simian virus 40
UCOE: Ubiquitous chromatin opening elements
UTR: Untranslated region
